# A multienergy computed tomography method without image segmentation or prior knowledge of X-ray spectra or materials

**DOI:** 10.1016/j.heliyon.2022.e11584

**Published:** 2022-11-15

**Authors:** Jiaotong Wei, Ping Chen, Bin Liu, Yan Han

**Affiliations:** Shanxi Key Laboratory of Signal Capturing & Processing, North University of China, Taiyuan 030051, People's Republic of China

**Keywords:** Computed tomography, Multienergy, Multivoltage projections, Blind decomposition, One scan, Prior knowledge

## Abstract

Many methods have been proposed for multienergy computed tomography (CT) imaging based on traditional CT systems. Usually, either prior knowledge of the X-ray spectra distribution or materials or the segmentation of the projection or reconstructed image is needed. To avoid these requirements, a multienergy CT method is proposed in this paper. A CT image can be seen as a linear combination of energy-dependent components and spatially dependent components. The latter components are the base images, while the former components are the coefficients. A blind decomposition model is constructed to decompose the multivoltage projections to obtain the base images and the energies. Multienergy CT images are computationally synthesized with the base images and the energies. Multivoltage projections can be acquired based on one scan with stepped voltages. X-ray scattering is considered an important factor in imaging errors and appears as a low-frequency signal. The variance is used to describe the low-frequency features and is minimized as the optimized objective function of the decomposition model. The solution of the model uses Karush–Kuhn–Tucker (KKT) conditions. In the experiments, the images reconstructed with the proposed method exhibit weak beam-hardening artifacts. Additionally, the X-ray energies of the different materials represented have small relative errors. Therefore, the reconstructed images have narrow energy intervals. This shows the effectiveness of the proposed method.

## 1 Introduction

Compared to conventional computed tomography (CT), multienergy CT can better distinguish between materials and has received increasing attention from researchers [1], [2]. There are many multienergy CT approaches, such as dual-energy CT, photon-counting detectors and multiple scanning [3], [4], [5].

Dual-energy CT can be seen as typical multienergy CT. The implementation methods include rapid kVp switching, multilayer detectors, dual sources and so on [6], [7]. According to its data processing method, dual-energy CT can be classified into two categories: projection domain and image domain methods [8]. Projection domain methods are based on projection decomposition. In the most typical type of method, high-energy projections and low-energy projections are joined with the concepts of X-ray imaging and decomposed into sinograms of two base materials. By using a conventional CT reconstruction algorithm for the sinograms of base materials, base material images can be obtained [8], [9]. Since the attenuation coefficients of the base materials are known, reconstruction images of different energy levels can be synthesized. The high-energy and low-energy projections must be consistent in terms of the projection angles [10]. In another category of projection domain methods, projection decomposition and image reconstruction are performed simultaneously through an iterative reconstruction framework [8]. Generally, prior knowledge of the X-ray spectrum is needed in projection domain methods, and estimation of X-ray spectra requires extra cost [11]. The image domain methods are based on reconstruction image decomposition [11], [12]. High-energy and low-energy projections are used to reconstruct high-energy images and low-energy images, respectively. For every pixel, the components of two base materials can be determined with both a high-energy image and a low-energy image. Then, two reconstructed images of the base materials are obtained. Polychromatic X-rays are equivalent to monochromatic X-rays with fixed energies in this method [13]. Since the equivalent energy is related to the imaging object, the fixed energy is inaccurate and cannot reflect the polychromatic character of practical X-rays [13], [14]. In recent years, deep learning has been developed in CT applications [15] and has been introduced into dual-energy CT imaging. Wenxiang Cong et al. proposed a modified residual neural network model to map single-spectrum CT images to virtual monoenergetic images, and the network training used clinical dual-energy CT data [16]. Daisuke Kawahara et al. used deep conditional generative adversarial networks to synthesize material decomposition images [17]. Y. Noda et al. proposed a deep-learning algorithm for dual-energy CT angiography to reduce iodine doses [18]. Wei Fang et al. used an image-domain deep learning framework to calculate effective atom number images with high-dual-energy CT [19].

Photon-counting CT uses a special detector for multienergy CT [20]. A photon-counting detector can determine the energy of a single X-ray photon. To set multiple energy thresholds for the detector, projections of different narrow energy intervals are acquired [21], [22]. Then, multienergy images are reconstructed. This is a promising method and is a future trend for multienergy CT. To date, there are still challenges for the widespread application of photon-counting detectors. The counting rate is not high enough to match the high flux of photons for CT imaging. K-escape events and crosstalk between adjacent pixels result in degradation of the energy resolution [22], [23], [24]. The fabrication of a large-area detector is also a challenge [25]. Based on photon-counting detectors, many imaging algorithms have been researched. Q Yang et al. proposed a superior method for a simultaneous algebraic reconstruction algorithm based on a prior rank, intensity and sparsity model that can reduce the artifacts and noise in the reconstructed images [26]. Korbinian Mechlem et al. proposed a statistical iterative algorithm for material image reconstruction that requires no detailed prior knowledge about the parameters of the imaging system [27]. Yidi Yao et al. proposed a dynamic dual-energy spectral CT method to improve the accuracy of multimaterial decomposition [28]. Xiaochuan Wu et al. improved fully convolutional DenseNets for multimaterial decomposition [29]. Hao Gong et al. developed a deep-learning method for the direct synthesis of low-energy virtual monoenergetic images [30]. Weiwen Wu et al. developed a U-net approach for image reconstruction with $L_{p}^{p}$-norm, total variation, residual learning, and anisotropic adaptation [31]. Doniyor Jumanazarov et al. proposed a reconstruction algorithm for sparse projections using vectorial total variation and material classification [32]. Xiang Chen et al. proposed a fourth-order nonlocal tensor decomposition model for spectral CT image reconstruction, and it achieved better performance than the compared methods [33].

In addition to the methods based on improving the hardware of CT systems, algorithm innovations have been researched to implement multienergy CT with conventional hardware. Le Shen et al. proposed an innovative scanning scheme for multienergy CT [34]. Buxin Chen et al. proposed a nonconvex optimization program and an algorithm for image reconstruction in multienergy CT. This algorithm can be used for nonstandard scan models without any hardware modification in existing CT systems [35]. They also researched a nonconvex primal-dual algorithm for image reconstruction in spectral CT [36]. These methods also require X-ray spectra. Renliang Gu et al. proposed a blind iterative reconstruction algorithm based on a statistical model for beam-hardening artifact correction without prior knowledge of the spectra or material [37]. Unfortunately, this algorithm can be used only for a single material object. Ping Chen et al. proposed multienergy CT methods using multivoltage projection sequence blind separation without prior knowledge of practical X-ray spectra [38], [39], [40], [41], [42]. Since these methods require full angle projection for every voltage, multiple scanning is implemented in imaging. Prior knowledge of the practical X-ray spectrum and attenuation characteristics of the materials is not needed.

However, we aim to develop a multienergy CT method that requires only one scan and no prior knowledge of the X-ray spectrum or imaging objects. Additionally, segmentation of the projections or reconstruction images is not expected. Sometimes, the image segmentation of directly reconstructed images, such as images of multimineral samples, is difficult [43]. To address this, a multienergy CT method for multimaterial objects is proposed. It is based on the blind decomposition of multivoltage projections with a stepped voltage potential scan. The projection acquisition of the method requires only one scan, inspired by the methods in references [34] and [35]. X-ray scattering is seen as an important factor that results in imaging errors. Since X-ray scattering is a low-frequency signal, we consider its change to be slow. The aim of decomposition is to minimize the variance of the decomposition errors. The solution uses the Karush-Kuhn–Tucker (KKT) conditions. The unknown variables of the energy coefficients of the physical effects are set as adaptive energy values. This is another innovation of the proposed method. The major contribution of this paper is to present a multienergy CT method that can be implemented in traditional CT systems with a potential one-time scan and requires no image segmentation or prior knowledge of the X-ray spectrum or materials.

The remainder of the paper is organized as follows. In Section 2, the blind decomposition model of multivoltage projection for multienergy CT imaging and the solution algorithm are introduced. In Section 3, the results of verification experiments and the evaluation are presented. In Section 4, a discussion is presented. Section 5 gives the conclusion.

## 2 Method

### 2.1 Polychromatic X-ray imaging model

The imaging model of polychromatic X-rays is

(1) $$I=\int_{0}^{E_{m}}I_{0}S(E)e^{-\int_{L}u(E,l)dl}dE,$$

where $I_{0}$ is the initial X-ray intensity and *I* is the X-ray intensity after the X-rays pass through the object. *E* indicates the X-ray energy, and $E_{m}$ is the maximal energy of the X-ray photons. *S*(*E*) is the normalized X-ray spectrum and satisfies

(2) $$\int_{0}^{E_{m}}S(E)dE=1,$$

where *L* is the X-ray path traveled. u(E,l) is the X-ray attenuation coefficient at location *l* when the X-ray energy is *E*. u(E,l) depends on the X-ray energy and the material. The physical effects resulting in X-ray attenuation include Rayleigh scattering, photoelectric effects, Compton scattering and electron-pair effects. u(E,l) can be decomposed as [34]

(3) $$u(E,l)=\sum_{k=1}^{K}u_{k}(E)b_{k}(l),$$

where *k* is the *k*-th physical effect. $u_{k}$(*E*) is the energy dependency component, and $b_{k}$(*l*) is the spatial dependency component. The spatial dependency components can be seen as the base images, and the energy dependency components are the weighting coefficients. Usually, industrial or medical CT systems, not accelerators, can consider only the photoelectric effect and Compton scattering [34], [44]. Formula (3) can be written as [14]

(4) $$u(E,l)=u_{1}(E)b_{1}(l)+u_{2}(E)b_{2}(l),$$

(5) $$u_{1}(E)=f_{\mathrm{ph}}(E)=\frac{1}{E^{3}},$$

(6) $$u_{2}(E)=f_{KN}(E)=\frac{2{\left(1+\alpha \right)}^{2}}{\alpha^{2}(1+2\alpha )}+\frac{\ln(1+2\alpha )}{\alpha }\times \left(\frac{1}{2}-\frac{1+\alpha }{\alpha^{2}}\right)-\frac{1+3\alpha }{{\left(1+2\alpha \right)}^{2}},$$

where α=E/511 keV. $f_{\text{ph}}(E)$ represents the energy coefficients of the photoelectric effect, and $f_{KN}(E)$ represents the energy coefficients of Compton scattering.

To divide the energy interval $\left[0,E_{m}\right]$ into *R* intervals, the boundary energy values are written as $E_{r}^{\prime }$ and

(7) $$0=E_{0}^{\prime }<E_{1}^{\prime }<\cdots <E_{r}^{\prime }<\cdots <E_{R}^{\prime }=E_{m}.$$

Then, formula (1) can be changed to

(8) $$\frac{I}{I_{0}}=\sum_{r=1}^{R}\int_{E_{r-1}^{\prime }}^{E_{r}^{\prime }}S(E)e^{-\int_{L}u(E,l)dl}dE.$$

By the first mean value theorem for integrals, there is an $E_{r}$ for

(9) $$\int_{E_{r-1}^{\prime }}^{E_{r}^{\prime }}S(E)e^{-\int_{L}u(E,l)dl}dE=e^{-\int_{L}u(E_{r},l)dl}\int_{E_{r-1}^{\prime }}^{E_{r}^{\prime }}S(E)dE,$$

and

(10) $$E_{r-1}^{\prime }<E_{r}<E_{r}^{\prime }.$$

We substitute formula (9) and formula (3) into formula (8), which gives

(11) $$\frac{I}{I_{0}}=\sum_{r=1}^{R}e^{-\sum_{k=1}^{K}u_{k}(E_{r})\int_{L}b_{k}(l)dl}\int_{E_{r-1}^{\prime }}^{E_{r}^{\prime }}S(E)dE.$$

Assuming that the imaging object has *T* pixels, the *k*-th base image is denoted as $X_{k}=(x_{k1},x_{k2},\ldots ,x_{kT}$). Then,

(12) $$\int_{L}b_{k}(l)dl=X_{k}P_{L}=\sum_{t=1}^{T}x_{kt}p_{tL}.$$

$P_{L}={\left(p_{1L},p_{2L},\ldots ,p_{TL}\right)}^{T}$ is the intersection length of the X-ray path *L* with the *t*-th pixel. We denote

(13) $$s_{r}=\int_{E_{r-1}^{\prime }}^{E_{r}^{\prime }}S(E)dE.$$

According to formula (2),

(14) $$1=\sum_{r=1}^{R}s_{r}.$$

We substitute formula (12) and formula (13) into formula (11); then,

(15) $$\frac{I}{I_{0}}=\sum_{r=1}^{R}s_{r}e^{-\sum_{k=1}^{K}u_{k}(E_{r})\left(X_{k}P_{L}\right)}.$$

### 2.2 Blind decomposition model of multivoltage projection

For formula (15), *I*/$I_{0}$ and $P_{L}$ are known. *I*/$I_{0}$ is the linear weighted sum of exponential terms with coefficients $s_{r}$. In typical CT imaging, the acquisition of $s_{r}$ depends on the spectrum estimation. If the coefficients $s_{r}$ are taken as unknown variables, formula (15) can be taken as a blind source separation model. This strategy is used here, and spectrum estimation is avoided. Therefore, *I*/$I_{0}$ is the observed signal, and the exponential terms represent the source signals. In fact, the base image $X_{k}$ is the essential source signal. Formula (15) is a special blind source separation model, and $X_{k}$ is the key unknown variable. According to the theory of blind source separation, the number of observed signals is generally no less than that of the source signals; otherwise, the problem will be more difficult [45]. More projections of different voltages are needed. Denoting the *I*/$I_{0}$ of the *m*-th projection of the *n*-th voltage as $f_{nm}$, the matrix form of formula (15) for imaging the *n*-th voltage is

(16) $$F_{n}=S_{n}e^{-U(E)XP_{n}},$$

where $F_{n}=(f_{n1},f_{n2},\ldots ,f_{nMn})$, $S_{n}=(s_{n1},s_{n2},\ldots ,s_{nR})$, $X={\left(x_{kt}\right)}_{KT}$ and $P_{n}={\left(p_{ntm}\right)}_{TMn}$. $E={\left(E_{1},E_{2},\ldots ,E_{R}\right)}^{T}$, $U(E)={\left(u_{rk}\right)}_{RK}$, and $u_{rk}=u_{k}(E_{r})$. $M_{n}$ represents the number of projections. $S_{n}$ is the weighting coefficient, and $s_{nr}$ is $s_{r}$ at the *n*-th voltage. $x_{kt}$ represents the *t*-th pixel of the *k*-th base image. $p_{\mathrm{ntm}}$ is the intersection length of the X-ray path of the *m*-th projection of the *n*-th voltage with the *t*-th pixel. When the photoelectric effect and Compton scattering are selected as the physical effects,

(17) $$U(E)=\left[\begin{array}{cc} f_{\mathrm{ph}}(E_{1})\; & f_{KN}(E_{1})\\ f_{\mathrm{ph}}(E_{2})\; & f_{KN}(E_{2})\\ \vdots \; & \vdots \\ f_{\mathrm{ph}}(E_{R})\; & f_{KN}(E_{R}) \end{array}\right].$$

Every row of *U*(**E**)*X* corresponds to a reconstruction image with a narrow energy interval.

Formula (15) is a theoretical imaging model, and interference factors, such as circuit noise, inconsistent responses of the detector cells and X-ray scattering, need to be considered in practical imaging. On the other hand, in formulas (8) to (12), $E_{r}$ is influenced by the X-ray path. In other words, the $E_{r}$ values for different projections should be different. Thus, $E_{r}$ is unknowable. Formula (16) needs to be changed to

(18) $$F_{n}=S_{n}e^{-U(E)XP_{n}}+\Delta F_{n},$$

where $\Delta F_{n}=(\Delta f_{n1},\Delta f_{n2},\ldots ,\Delta f_{nMn})$ is the error of imaging model (16). $S_{n}$, *X*, *U*(*E*) and $\Delta F_{n}$ are all unknown variables. Referring to the solution of the blind separation model, model (18) needs to be transformed into an optimization model to obtain the key variables *X* and *U*(**E**). The most crucial problem is to describe the nature of $\Delta F_{n}$ with an appropriate function as the optimized objective function. Traditionally, X-ray scattering is corrected or ignored, and then, the optimized objective function minimizes the sum of the squared errors of all $\Delta F_{n}$ as

(19) $$\min\sum_{n=1}^{N}{\left\|F_{n}-S_{n}e^{-U(E)XP_{n}}\right\|}_{2}^{2},$$

where the subscript “2” indicates the 2-norm of the vector and the superscript “2” indicates the square [1], [11], [28], [38], [42]. This model can be derived by assuming that the projection errors come from many factors, which can be seen for random variables with 0 expectation. Under this assumption, the projection errors obey a Gaussian distribution. With maximum likelihood estimation, the optimization model is model (19). If X-ray scattering cannot be ignored, the mean value of $\Delta F_{n}$ is considered the expectation of X-ray scattering. This is the result of other error factors being seen as random variables with 0 expectation. From this, the residual after the average is subtracted from $\Delta F_{n}$ satisfy model (19). This can be expressed as

(20) $$\min\sum_{n=1}^{N}{\left\|\Delta F_{n}-mean\left(\Delta F_{n}\right)\right\|}_{2}^{2},$$

where *mean*($\Delta F_{n}$) is the average of $\Delta F_{n}$. Since the variance of $\Delta F_{n}$ is

(21) $$\frac{1}{M_{n}}{\left\|\Delta F_{n}-mean\left(\Delta F_{n}\right)\right\|}_{2}^{2},$$

the square of ${\left\|\Delta F_{n}-\text{mean}(\Delta F_{n})\right\|}_{2}$ is the $M_{n}$ multiple of the variance of $\Delta F_{n}$. Ignoring the coefficient $M_{n}$, the variance of $\Delta F_{n}$ can be used to replace the square of ${\left\|\Delta F_{n}-\text{mean}(\Delta F_{n})\right\|}_{2}$ in model (20). On the other hand, using the variance of $\Delta F_{n}$ to replace the square of ${\left\|\Delta F_{n}\right\|}_{2}$ in model (19) can be derived from signal analysis. According to references [46], [47], X-ray scattering is an important factor influencing $\Delta F_{n}$. X-ray scattering is a low-frequency signal [47], [48], and $I_{0}$ is larger than 1. Thus, $\Delta F_{n}$ should exhibit a low-frequency characteristic. In other words, the values of $\Delta F_{n}$ change slowly. The mean value of $\Delta F_{n}$ is considered the approximation of X-ray scattering. Therefore, the variance of $\Delta F_{n}$ is used as the optimized objective function. Since there are many projections of different angles in CT imaging, the optimized model for solving model (18) is written as

(22) $$\min\;G=\sum_{n=1}^{N}\sum_{j=1}^{J_{n}}\left(\mathrm{var}\;\left(\Delta F_{nj}\right)\right),$$

where *j* is the index of the projection angles and $J_{n}$ is the total number of angles at the *n*-th voltage. $\Delta F_{nj}$ represents the $\Delta F_{n}$ of model (18) at the *j*-th angle of the *n*-th voltage. var(•) means the variance of “•”. If there is no X-ray scattering, model (22) degrades into model (19).

Theoretically, if there is no X-ray scattering, half of the imaging errors are less than 0, and the rest are greater than 0. Since the intensity of X-ray scattering is a positive value or 0, more of the imaging errors are greater than 0, but the proportion is unknown. For this, we select the initialization values of $S_{n}$, *E* and *X* to make all the initial errors $\Delta F_{nj}$ greater than 0.

Considering constraint formula (14), the optimized model (22) can be written as

(23) $$\begin{array}{cc} \min\; & G=\sum_{n=1}^{N}\sum_{j=1}^{J_{n}}\left(\mathrm{var}\;\left(\Delta F_{nj}\right)\right)\\ s.t.\; & \left\{ \begin{array}{c} \sum_{r=1}^{R}s_{nr}=1\\ S_{n}\geq 0,\;E>0,\;X\geq 0 \end{array}\right. \end{array}.$$

$S_{n}$, **E** and *X* are unknown variables. If *S*_*n*0_, $E_{0}$ and $X_{0}$ are the optimal solutions, then *H* satisfies

(24) $$H=S_{n0}e^{-U(E_{0})X_{0}P_{n}}.$$

Essentially, this is an equation set. Every element of *H* implies an equation for *S*_*n*0_, *E*_*r*0_ and $X_{0}$. To reduce the multiplicity of the solution, the number of equations should be greater than the number of unknown variables; i.e.,

(25) $$\sum_{n=1}^{N}\sum_{j=1}^{J_{n}}M_{nj}>NR+R+KT.$$

Generally, *T* is much larger than *N* and *R*. It can consider only $M_{nj}$ and *KT*. Theoretically, equation (25) also contributes to reducing the possible bias produced by equation (22), which controls only smoothness.

### 2.3 Solving

The nonnegative matrix factorization solution is referred since model (23) is a blind separation model with nonnegative constraints [49]. To refer to the nonnegative matrix factorization algorithm, the equality constraints regarding *s*_*n*r_ are temporarily ignored. The KKT conditions are used to derive the iterative solution formulas. The detailed process is presented in Appendix A. The iterative formulas of $S_{n}$, **E** and *X* are

(26) $$S_{n}=S_{n}⊙\frac{\left(\left(S_{n}e^{-U(E)XP_{n}}\right)O_{nM}+\left(F_{n}\right)T_{nM}D_{nM}\right){\left(e^{-U(E)XP_{n}}\right)}^{T}}{\left(\left(F_{n}\right)O_{nM}+\left(S_{n}e^{-U(E)XP_{n}}\right)T_{nM}D_{nM}\right){\left(e^{-U(E)XP_{n}}\right)}^{T}},$$

(27) $$E=E⊙\frac{\left(\sum_{n=1}^{N}\left(\left(S_{n}^{T}\left(\left(S_{n}e^{-U(E)XP_{n}}\right)O_{nM}+\left(F_{n}\right)T_{nM}D_{nM}\right)\right)⊙\left(e^{-U(E)XP_{n}}\right)\right){\left(XP_{n}\right)}^{T}\right)U^{\prime }(E)}{\left(\sum_{n=1}^{N}\left(\left(S_{n}^{T}\left(\left(F_{n}\right)O_{nM}+\left(S_{n}e^{-U(E)XP_{n}}\right)T_{nM}D_{nM}\right)\right)⊙\left(e^{-U(E)XP_{n}}\right)\right){\left(XP_{n}\right)}^{T}\right)U^{\prime }(E)},$$

(28) $$X=X⊙\frac{\sum_{n=1}^{N}\left({\left(U(E)\right)}^{T}\left(\left(S_{n}^{T}\left(\left(Se^{-U(E)XP_{n}}\right)O_{nM}+\left(F_{n}\right)T_{nM}D_{nM}\right)\right)⊙\left(e^{-U(E)XP_{n}}\right)\right)\right)P_{n}^{T}}{\sum_{n=1}^{N}\left({\left(U(E)\right)}^{T}\left(\left(S_{n}^{T}\left(\left(F_{n}\right)O_{nM}+\left(S_{n}e^{-U(E)XP_{n}}\right)T_{nM}D_{nM}\right)\right)⊙\left(e^{-U(E)XP_{n}}\right)\right)\right)P_{n}^{T}}.$$

The symbol ‘⊙’ represents the Hadamard product of matrices, and it means the corresponding elements of two matrices are to be multiplied. $U^{\prime }$(**E**) is the derivative of *U*(**E**) with respect to **E**:

$$U^{\prime }(E)=\left[\begin{array}{cc} f_{\mathrm{ph}}^{\prime }(E_{1})\; & f_{KN}^{\prime }(E_{1})\\ f_{\mathrm{ph}}^{\prime }(E_{2})\; & f_{KN}^{\prime }(E_{2})\\ \vdots \; & \vdots \\ f_{\mathrm{ph}}^{\prime }(E_{R})\; & f_{KN}^{\prime }(E_{R}) \end{array}\right].$$

*f*_ph_′(*E*) and *f*_KN_′(*E*) are the derivatives of *f*_ph_(*E*) and *f*_KN_(*E*), respectively.

$O_{nM}$ is a matrix with $\sum_{g=1}^{J_{n}}M_{ng}$ rows and $\sum_{g=1}^{J_{n}}M_{ng}$ columns, as follows:

$${\left(O_{nM}\right)}_{row,column}=\left\{ \begin{array}{c} \frac{1}{M_{nj}},row=column=m+\sum_{g=1}^{j-1}M_{ng},m=1,2,\cdots ,M_{nj}\\ 0,others \end{array}\right..$$

$D_{nM}$ is a matrix with $\sum_{g=1}^{J_{n}}M_{ng}$ rows and $\sum_{g=1}^{J_{n}}M_{ng}$ columns, as follows:

$${\left(D_{nM}\right)}_{row,column}=\left\{ \begin{array}{c} \frac{1}{M_{nj}^{2}},row=column=m+\sum_{g=1}^{j-1}M_{ng},m=1,2,\cdots ,M_{nj}\\ 0,others \end{array}\right..$$

$T_{nM}$ is a matrix with $\sum_{g=1}^{J_{n}}M_{ng}$ rows and $\sum_{g=1}^{J_{n}}M_{ng}$ columns, as follows:

$$T_{nM}=\left[\begin{array}{ccccc} 1_{M_{n1}\times M_{n1}}\; & 0\; & \cdots \; & 0\; & 0\\ 0\; & 1_{M_{n2}\times M_{n2}}\; & \cdots \; & 0\; & 0\\ \vdots \; & \vdots \; & \ddots \; & \vdots \; & \vdots \\ 0\; & 0\; & \cdots \; & 1_{M_{n(J_{n}-1)}\times M_{n(J_{n}-1)}}\; & 0\\ 0\; & 0\; & \cdots \; & 0\; & 1_{M_{nJ_{n}}\times M_{nJ_{n}}} \end{array}\right].$$

$1_{M_{nj}\times M_{nj}}$is a matrix with $M_{nj}$ rows and $M_{nj}$ columns, every element of which is 1, as follows:

$$1_{M_{nj}\times M_{nj}}=\left[\begin{array}{ccccc} 1\; & 1\; & \cdots \; & 1\; & 1\\ 1\; & 1\; & \cdots \; & 1\; & 1\\ \vdots \; & \vdots \; & \ddots \; & \vdots \; & \vdots \\ 1\; & 1\; & \cdots \; & 1\; & 1\\ 1\; & 1\; & \cdots \; & 1\; & 1 \end{array}\right].$$

Regarding the constraint of $s_{nr}$, i.e., formula (14), used to imitate nonnegative matrix factorization, the sum of the elements of $S_{n}$ must be normalized after every iteration.

### 2.4 Initialization of the iterative solution

The iterative solution must be initialized.

For $E_{r}$, the initialization value is the middle energy of the *r*-th energy interval, and the *R* energy intervals are equally divided from 0 to $E_{m}$.

For $s_{nr}$, the values for the middle energies are larger than the values for the high and low ends of the energy spectrum. A parabolic formula is used to describe the continuous spectrum as

(29) $$S(E)=E\left(E_{m}-E\right).$$

To discretize *S*(*E*) and to normalize the discrete values, the initialization value of $s_{nr}$ is calculated as

(30) $$s_{nr}=\frac{\int_{E_{r-1}^{\prime }}^{E_{r}^{\prime }}E\left(E_{m}-E\right)dE}{\int_{0}^{E_{m}}E\left(E_{m}-E\right)dE},$$

where $E_{m}$ is the maximum photon energy of the *n*-th voltage.

$x_{kt}$ is set as

(31) $$x_{kt}=\frac{\frac{1}{K}y_{t}}{u_{k}\left(E_{\overset{¯}{r}}\right)},\overset{¯}{r}=\left[\frac{R}{2}+0.5\right],$$

where $Y=(y_{1},y_{2},\ldots ,y_{T})$ is a directly reconstructed image with the projections of *n* voltages.

The initial values of $E_{r}$, $s_{nr}$ and $x_{kt}$ may not make all initial errors $\Delta f_{\mathrm{njm}}$ greater than 0. If so, we update *r* with

(32) $$\overset{¯}{r}=\overset{¯}{r}+1$$

until every initial error $\Delta f_{\mathrm{njm}}$ is greater than 0.

### 2.5 Algorithm pseudocode

The pseudocode of the algorithm for solving model (23) is shown below.

**Figure:**
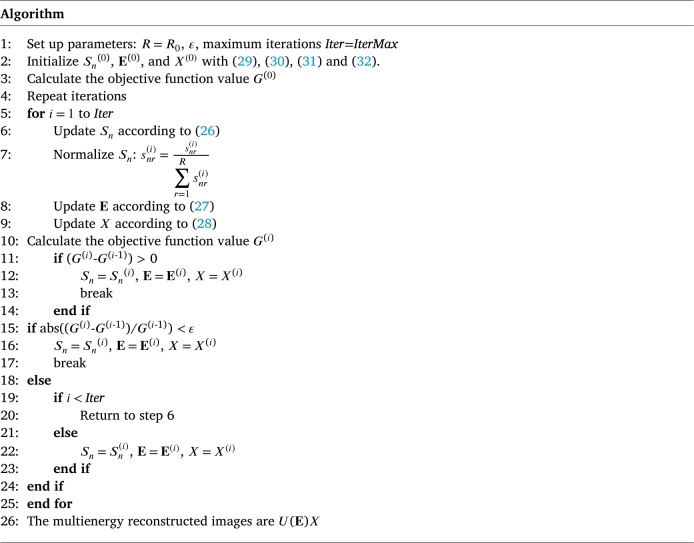

## 3 Results

**Figure 1:**
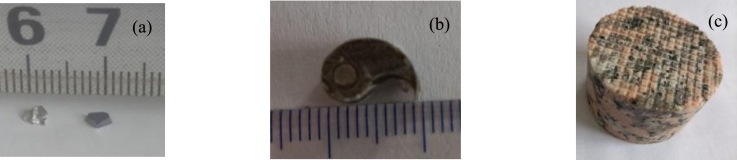
Three samples. (a) Aluminous and silicic fragments. (b) Steel cylinder and part of a titanium alloy cylinder. (c) Columnar marble.

**Figure 2:**
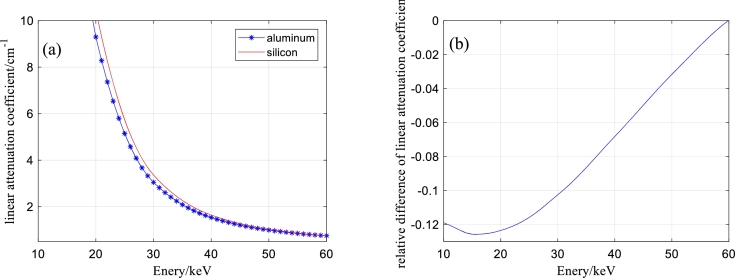
Attenuation coefficients of aluminum and silicon and their differences. (a) Attenuation coefficients of aluminum and silicon. (b) Relative differences in the attenuation coefficients.

**Figure 3:**
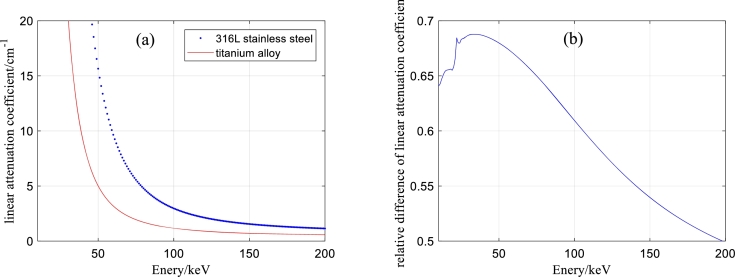
Attenuation coefficients of 316L stainless steel and titanium alloy and their differences. (a) Attenuation coefficients of 316L stainless steel and titanium alloy. (b) Relative differences in the attenuation coefficients.

The three multimaterial samples in Fig. 1 [41] are used to verify the proposed method. One sample is composed of aluminous and silicic fragments, and the relative differences in the attenuation coefficients of the two materials are shown in Fig. 2. The second sample is composed of 316L stainless steel and titanium alloy (Ti-6Al-4V), and the relative differences in the attenuation coefficients of the two materials are shown in Fig. 3. All the attenuation coefficients are calculated based on the material components. The attenuation coefficients of the material components are obtained by cubic spline interpolation of the attenuation coefficients of the National Institute of Standards and Technology (NIST). The third sample is cylindrical marble with unknown impurities. Its diameter is approximately 50 mm.

A YXLON FF20 CT system in microfocus mode is used to obtain the projections. This system is located at the Shanxi Key Laboratory of Signal Capturing & Processing of the North University of China. The flat panel detector has 1122 ×1122 cells, and the size of a cell is 0.127 mm × 0.127 mm. A cell size of 0.127 mm is used as the unit length for the related calculation. The gray value of the reconstructed images represents the attenuation coefficients. To calculate the X-ray energy of the reconstruction images represented, the attenuation coefficients are calculated as the gray values multiplied by 10/0.127, and the unit is cm^−1^.

**Figure 4:**
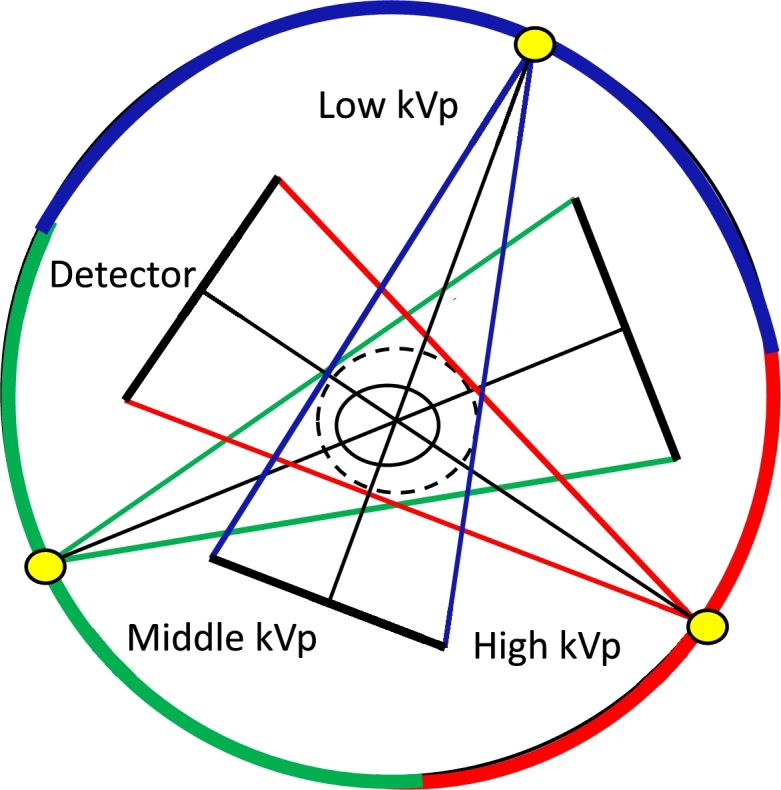
Model of projection acquisition with three voltages.

Model (23) requires multivoltage projections, and projection consistency among the different voltages is not needed. In other words, the projections of one angle can be acquired with multiple voltages or with only one voltage. A model divides all the projection angles into different angle intervals, and different voltages are used for different angle intervals. Only one scan is needed in this model. One example is the stepped voltage potential scan proposed by Le Shen [34], shown in Fig. 4. It is used in the experiments of this paper. The three angle intervals are equant.

### 3.1 Experiment on a sample composed of aluminous and silicic fragments

#### 3.1.1 Imaging parameters

The aluminous and silicic fragments are combined with woolen yarn. The sizes of the fragments are in millimeters.

The distance from the X-ray source to the detector is 780.577 mm. The distance from the X-ray source to the object is 99.999 mm. The range of the projection angles is (0, 360], and the projection interval is 1 degree.

To compare the reconstructed images, all projections of the 360 angles are acquired for every voltage. The three voltages are 40 kV, 50 kV and 60 kV, and the corresponding projections of angle intervals 1–120, 121–240, and 241–360 are selected for the proposed method. The tube currents are all 60 μA.

#### 3.1.2 Reconstructed images and comparison

A 2-dimensional (2D) slice through the center of the sample is reconstructed. In the direct reconstruction and the compared reconstruction, the iterative algebraic reconstruction algorithm (ART) is used, and the step length is 0.01. The number of iterations is 120. The gray value of the reconstructed images is forced to be no less than 0. To reduce the noise, a 5×5 median filter is used for all the reconstructed images. These parameters are also used in other experiments in this paper. The size of the reconstructed images is set to 256×256.

**Figure 5:**
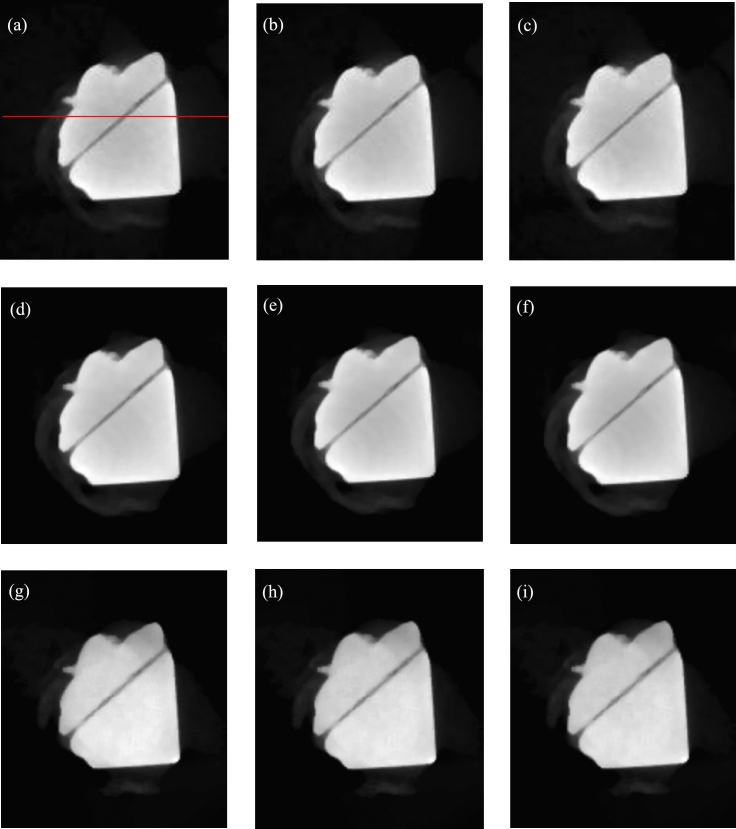
Reconstructed images of sample composed of aluminous and silicic fragments. (a)-(c) Directly reconstructed images of 40 kV, 50 kV and 60 kV. (d)-(f) Second, fourth and sixth images reconstructed with decomposed projections obtained by method of reference [42]. (g)-(i) Second, fourth and sixth images reconstructed with decomposed projections obtained by method of this paper.

**Figure 6:**
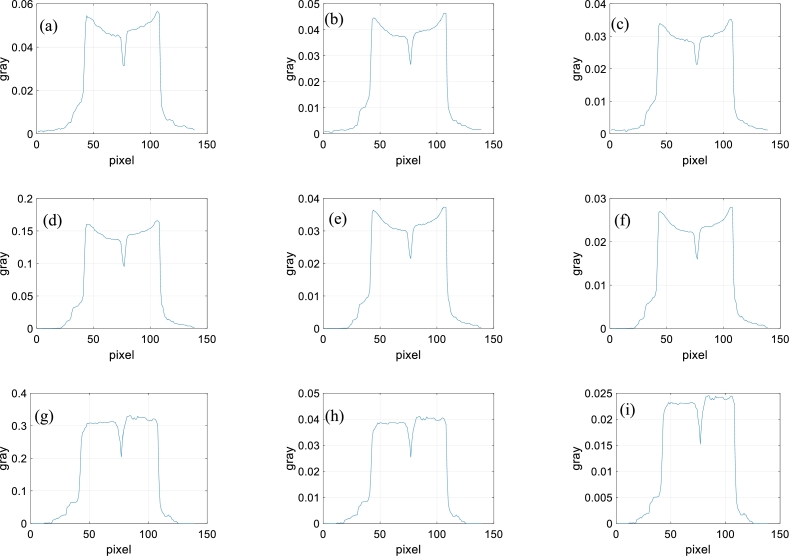
Gray values. (a)-(i) Gray values of the same middle row for the images (a)-(i) of Fig. 5.

The direct reconstruction images are shown in the first line of Fig. 5. The gray values of the red line correspond to the first line of Fig. 6.

To compare the results, the method of reference [42] is selected since it requires no prior knowledge of the practical X-ray spectra or component materials. We write code based on the paper. Some reconstruction images are shown in the second line of Fig. 5. The gray values of the red line correspond to the second line of Fig. 6.

In the proposed method, the initial energy interval width is set to 10 keV. The parameters are set to R=6, *ε*=10^−3^, and *IterMax*=100. Some reconstructed images are shown in the third line of Fig. 5. The gray values of the red line correspond to the third line of Fig. 6.

If a reconstructed image has narrow energy intervals, it should have two features. First, the energies of the attenuation coefficients of the different materials represented should be the same in one CT image. Second, the same materials should have approximately identical gray values in the reconstructed image. In other words, the beam-hardening artifacts should be as weak as possible.

From Fig. 5, for the directly reconstructed images and the images reconstructed with the method of reference [42], the center of the sample is darker than the surroundings. Hardening artifacts can be observed. This is not obvious for the images reconstructed with the proposed method. From the gray values of Fig. 6, the hardening artifacts in the images reconstructed by the proposed method are weak, the weakest among those of the three reconstruction methods. The hardening artifacts are obvious in the directly reconstructed images and the images reconstructed with the method of reference [42].

**Figure 7:**
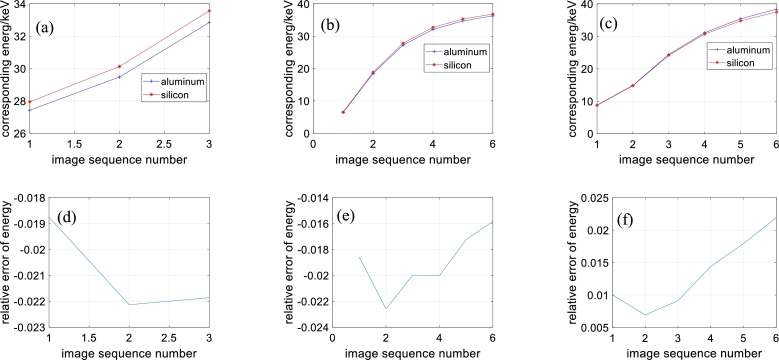
Energies of aluminous and silicic materials and relative errors. (a)-(c) Energies of directly reconstructed images, reconstructed images obtained by method of reference [42] and reconstructed images obtained by method of this paper. (d)-(e) Relative energy errors of directly reconstructed images, reconstructed images obtained by method of reference [42] and reconstructed images obtained by method of this paper.

According to the average gray values of the aluminous and silicic materials, we calculate their corresponding X-ray energies. The energies of the attenuation coefficients of aluminous and silicic fragments from different methods and the relative errors are presented in Fig. 7. The maximal X-ray energy difference of the aluminous and silicic fragments is close to 2%. The mean X-ray energy difference of the reconstruction images of the proposed method is the lowest among those of the three reconstruction methods. Combined with the results of the hardening artifacts, the reconstructed images of the proposed method have narrow energy intervals.

Since the complexity analysis of the proposed method is beyond the present skills of the authors, a comparison of the run times of the different methods is given. The run times of the different methods are shown in Table 1, Table 2, Table 3. The C++ code of the reconstruction process is implemented in Visual Studio. The code of the projection decompositions for the method of reference [42] and the proposed method is written in MATLAB. A computer with a 4-core Intel CPU and 8 gigabytes of memory is used. The CPU is an Intel Core i5-7300HQ CPU with a 2.5 GHz processor.

**Table 1:** Running times of direct reconstruction (in sec.)

Reconstruction algorithm (C++)	ART
Number of iterations	120
Total time for the three voltages	2020
Mean time for every reconstructed image	612.7

**Table 2:** Running times of the method in reference [42] (in sec.)

Projection decomposition (MATLAB)	Number of effective iterations	6
	Running times	0.89
Reconstruction (C++)	Reconstruction algorithm	ART
	Number of iterations	120
	Total time for the six decomposed projections	3988
Total running time		3988.89
Mean time for one reconstructed image		664.82

**Table 3:** Running times of the proposed method (in sec.)

Initial reconstruction to produce the initial values of *X* (C++)	Reconstruction algorithm	ART
	Number of iterations	120
	Running times	672
Computation of the projection matrix (MATLAB)		56.39
Number of effective iterations (MATLAB)		11
Running time of the iterations		7.12
Total running time		735.51
Mean time for one reconstructed image		113.19

From Table 1, Table 2, Table 3, most of the running time is consumed by the reconstruction process. The running time for projection decomposition can almost be ignored. Comparing the mean times, that of the proposed method is the lowest. If only one image is reconstructed, the largest mean time is that of the proposed method, since the initial value of *X* is still needed.

### 3.2 Experiment on a sample composed of steel and titanium alloy

#### 3.2.1 Imaging parameters

The sample is composed of one steel cylinder with a diameter of 2.5 mm and part of a titanium alloy cylinder with a diameter of 10 mm.

The distance from the X-ray source to the detector is 779.577 mm. The distance from the X-ray source to the object is 200.000 mm. The range of the projection angles is (0, 360], and the projection interval is 1 degree.

To compare the reconstructed images, all projections of the 360 angles are acquired for every voltage. The three voltages are 160 kV (40 μA), 170 kV (38 μA) and 180 kV (38 μA), and the corresponding projections of the angle intervals 1–120, 121–240, and 241–360 are selected for the proposed method.

#### 3.2.2 Reconstructed images

A 2D slice through the center of the sample is reconstructed. The size of the reconstructed images is set to 256×256.

**Figure 8:**
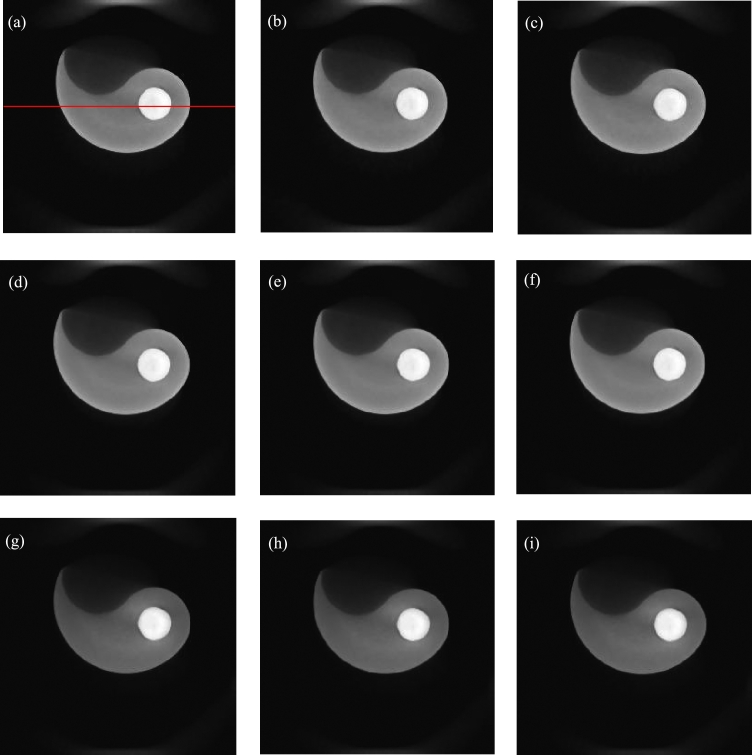
Reconstructed images of sample composed of steel and titanium alloy. (a)-(c) Directly reconstructed images of 160 kV, 170 kV and 180 kV. (d)-(f) Sixth, twelfth and eighteenth images reconstructed with decomposed projections obtained by method of reference [42]. (g)-(i) Sixth, twelfth and eighteenth images reconstructed with decomposed projections obtained by method of this paper.

**Figure 9:**
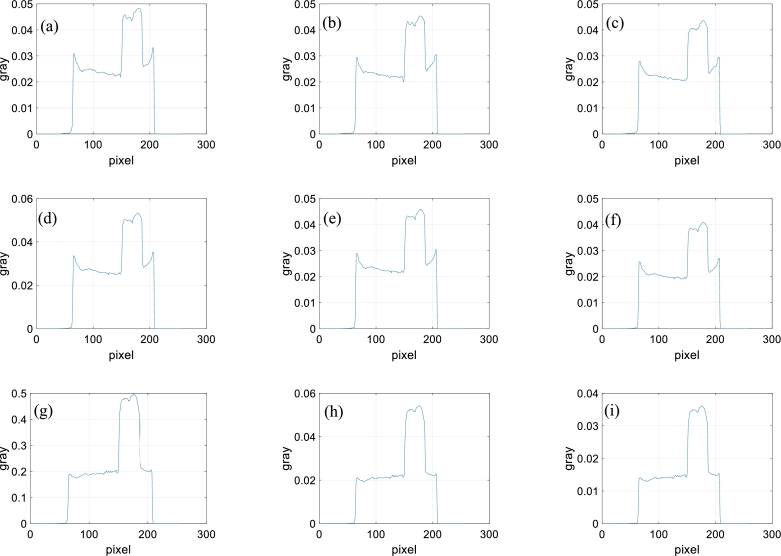
Gray values. (a)-(i) Gray values of the same middle row for the images (a)-(i) of Fig. 8.

The direct reconstruction images are shown in the first line of Fig. 8. The gray values of the red line correspond to the first line of Fig. 9.

Some reconstruction images obtained by the method of reference [42] are shown in the second line of Fig. 8. The gray values of the red line correspond to the second line of Fig. 9. Since the Spectrum GUI_1.03 software used in reference [42] cannot produce X-ray spectra for voltages higher than 140 kV, the initial values of the discrete spectrum are set to those of the method proposed in this paper.

In the proposed method, the initial energy interval width is set to 10 keV. The parameters are set to R=18, $\varepsilon =10^{-3}$, and *IterMax*=500. Some reconstructed images are shown in the third line of Fig. 8. The gray values of the red line correspond to the third line of Fig. 9.

From Fig. 8 and Fig. 9, similar to the previous experiment in Section 3.1, the hardening artifacts in the reconstructed images of the direct reconstruction and compared methods are obviously larger than those in the reconstructed images of the proposed method. In Fig. 8, for the directly reconstructed images and the images reconstructed with the method of reference [42], the outer edge is obviously brighter than the inside region. This phenomenon is almost invisible in the images reconstructed with the proposed method.

**Figure 10:**
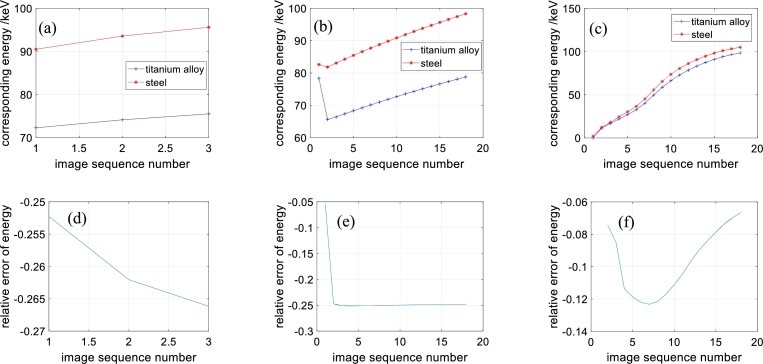
Energies of steel and titanium alloy and relative errors. (a)-(c) Energies of directly reconstructed images, reconstructed images obtained by method of reference [42] and reconstructed images obtained by method of this paper. (d)-(e) Relative energy errors of directly reconstructed images, reconstructed images obtained by method of reference [42] and reconstructed images obtained by method of this paper.

The energies of the steel and titanium alloy obtained from different methods and the relative errors are presented in Fig. 10.

From Fig. 10, in the images reconstructed by direct reconstruction, the relative errors of the energies of the steel and titanium alloy are larger than 25%. In the reconstructed images of the compared method, the relative errors of the energies of steel and titanium alloy are approximately 25%, except for that of the first image. In the reconstructed images of the proposed method, the relative errors of the energies of steel and titanium alloy are smaller than 13%.

Overall, the proposed method produces reconstructed images with the narrowest energy intervals among the three methods.

### 3.3 Experiment on a marble sample

The distance from the X-ray source to the detector is 780.000 mm. The distance from the X-ray source to the object is 570.000 mm. The range of the projection angles is (0, 360], and the projection interval is 1 degree.

To compare the reconstructed images, all projections of the 360 angles are acquired for every voltage. The three voltages are 160 kV (40 μA), 170 kV (40 μA) and 180 kV (35 μA), and the corresponding projections of the angle intervals 1–120, 121–240 and 241–360 are selected for the proposed method.

A 2D slice through the center of the sample is reconstructed. The size of the reconstructed images is set to 500×500.

**Figure 11:**
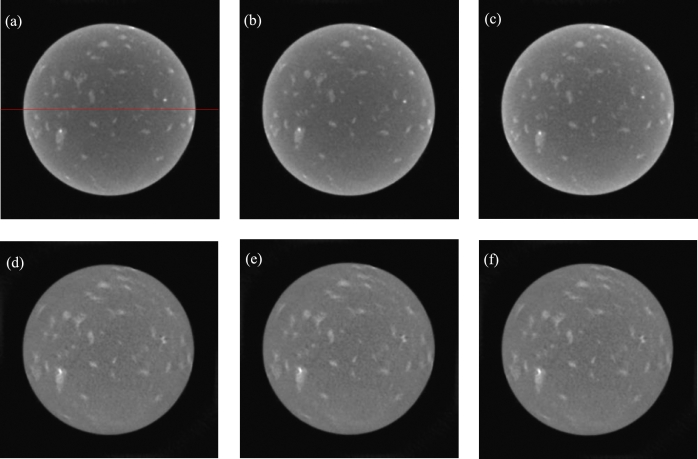
Reconstructed images of the marble sample. (a)-(c) Directly reconstructed images of 160 kV, 170 kV and 180 kV. (d)-(f) Sixth, twelfth and eighteenth images reconstructed with decomposed projections obtained by method of this paper.

**Figure 12:**
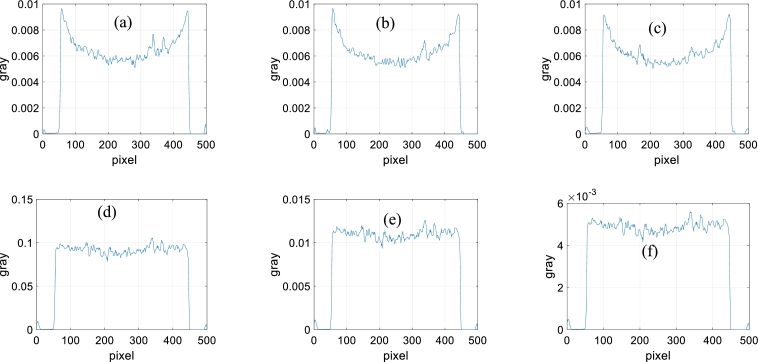
Gray values. (a)-(f) Gray values of the same middle row for the images (a)-(f) of Fig. 11.

The images reconstructed by direct reconstruction are shown in the first line of Fig. 11. The gray values of the red line correspond to the first line of Fig. 12.

In the proposed method, the initial energy interval width is set to 10 keV. The parameters are set to R=18, $\varepsilon =10^{-3}$, and *IterMax*=500. Some reconstructed images are shown in the second line of Fig. 11. The gray values of the red line correspond to the second line of Fig. 12.

From Fig. 11, the marble sample contains at least three components. The first component is the base material. The second component is represented by a bright dot. The third component is the dispersed gray regions. From Fig. 11, in the directly reconstructed images, the edge regions are brighter than the central regions. This is not shown in the images reconstructed by the proposed method. From Fig. 12, the directly reconstructed images exhibit obvious hardening artifacts. The hardening artifacts of the images reconstructed by the proposed method are inconspicuous.

From the perspective of the hardening artifacts, the images reconstructed by the proposed method have narrow energy intervals.

## 4 Discussion

A multienergy CT method for multimaterial objects is proposed in this paper. This method is based on a blind source separation variant of multivoltage projections with a stepped voltage potential scan. Compared with multienergy CT methods that require photon-counting detectors, such as the methods of references [25], [26], [27], [29], [30], [33], this method requires no hardware changes in traditional CT systems except voltage control. Compared with most multienergy CT methods based on traditional CT systems, the proposed method has four advantages. First, it requires no prior knowledge of the X-ray spectra; prior knowledge is required in many multienergy CT methods [34], [35]. This is achieved by the blind decomposition of projections. Second, it requires no prior knowledge of the materials. This is achieved by replacing the base materials with base physical effects. The energy dependency components of the physical effects are extracted and represented by the X-ray energy. Third, the proposed method requires no image segmentation of the projections or reconstructed images. Fourth, this method can be implemented with only one scan by stepped voltages. In other words, it does not require projections of different voltages to be consistent, while this is needed in the methods of references [38], [39], [40], [41], [42]. This advantage is the result of image-domain decomposition.

Three multimaterial samples were used in the experiments. They represented different imaging scenarios. The experiment on the aluminum and silicon material represented imaging objects containing materials with proximate attenuation characteristics. This shows that the proposed method can be used to distinguish materials with proximate attenuations. The experiment on the steel and titanium alloy shows the effectiveness of the proposed method for objects with materials with obvious differences in attenuation characteristics. The marble experiment shows that the proposed method can function without image segmentation of the projections or reconstructed images. For natural mineral samples, the components usually have blurry edges, and image segmentation is inapplicable. Since the authors are not qualified to perform CT imaging on biomedical materials, testing on relevant biomedical objects was not performed. From the experiments, the proposed method achieves the goal of multienergy CT imaging. From the experiments on the first and second samples, the reconstructed images of the proposed method have small relative error differences, which agree with a characteristic of CT images with narrow energy intervals. Additionally, the reconstructed images have weak hardening artifacts, which agree with another characteristic of CT images with narrow energy intervals.

However, some problems also exist for the proposed method. First, the projection decomposition model needs to be improved. From Fig. 7 (c) and Fig. 10 (c), the maximum X-ray energies of the reconstructed images represented are lower than 2/3 of the theoretical maximum. The X-ray energies are low overall. On the other hand, the relative errors of the X-ray energies obviously increase from Fig. 7 (c) to Fig. 10 (c) as the relative differences in the attenuation coefficients of the materials increase. Intuitively, from the structure and attenuation characteristics, the X-ray scattering of the first sample should be smoother than that of the second sample. These two points imply that model (23) is a rough mathematical description of the error $\Delta F_{n}$ of model (18). A more rigorous model is needed. The second problem concerns the algorithm convergence conditions. The optimization procedure is the entire imitation of the nonnegative matrix factorization algorithm. They should have similar convergence. The authors aim to solve this problem in future research. The third problem is the initial value selection. This is related to the second problem. The initial values influence convergence. Additionally, as model (23) is a nonconvex problem, the solution is influenced by the initial values.

**Figure 13:**
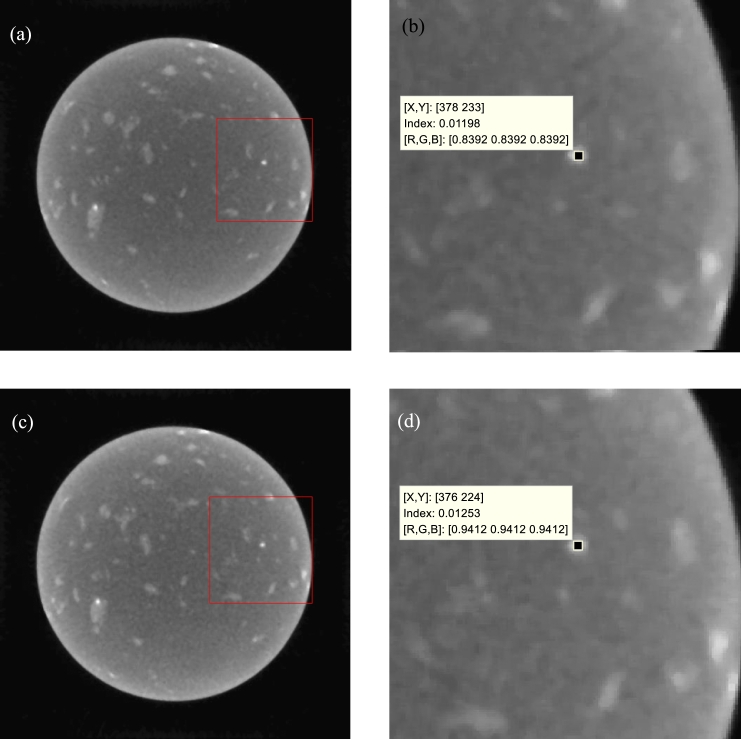
Location error in directly reconstructed images. (a)-(b) The image of 170 kV and its part. (c)-(d) The image of 180 kV and its part.

Another issue concerns the precision of the CT system hardware. From Fig. 11, the details of the images reconstructed with the proposed method are blurrier than those of the directly reconstructed images. This occurs because the projections of the different voltages we used are acquired three times, not once. The projection angles have errors for different voltages. This can be avoided with the use of one scan. For example, the location error of a detail in the directly reconstructed images is shown in Fig. 13. The left images correspond to 170 kV and 180 kV. The right images are part of the left images. The center coordinates of an especially bright dot are (378, 233) in the 170 kV image and (376, 224) in the 180 kV image.

From the above analysis, future work should focus on the following aspects. One is to build a more rigorous model of projection decomposition. This will make the distribution of the X-ray energies of the reconstructed images more reasonable. Another is to research algorithm convergence and the initial value selection. The third task is to analyze the measurement accuracy of the projection decomposition model. We only present the accuracy for the experiments on the given samples. Theoretical accuracy analysis is presently a difficulty for the authors. We aim to learn error theory to analyze the accuracy of the proposed method.

## 5 Conclusion

In this paper, a multienergy CT method without the need for prior knowledge that can be used for multimaterial objects is proposed. The method is based on the decomposition of multivoltage projections. It requires no prior knowledge of the X-ray spectra or materials. Additionally, it requires no image segmentation of projections or reconstructed images. Multivoltage projections can be acquired based on a one-step voltage scan. Three multimaterial samples representing different imaging scenarios show the effectiveness of the proposed method. Future research should focus on improving the model of projection decomposition, algorithm convergence, initial value selection and accuracy analysis of the decomposition model.

## Declarations

### Author contribution statement

Jiaotong Wei: Conceived and designed the experiments; Performed the experiments; Analyzed and interpreted the data; Contributed reagents, materials, analysis tools or data; Wrote the paper.

Ping Chen & Yan Han: Conceived and designed the experiments; Bin Liu: Contributed reagents, materials, analysis tools or data.

### Funding statement

This work was supported by 10.13039/501100001809National Natural Science Foundation of China (No. 62201520, 61871351, 61801437, 61971381 & 62001429), 10.13039/501100020771Natural Science Foundation for Young Scientists of Shanxi Province (No. 201901D211247, 201901D211250).

### Data availability statement

Data will be made available on request.

### Declaration of interests statement

The authors declare no conflict of interest.

### Additional information

No additional information is available for this paper.

## Appendix A

The detailed expression of *G* is

(A.1) $$G=\sum_{n=1}^{N}\sum_{j=1}^{J_{n}}\left(\frac{1}{M}\sum_{m=1}^{M_{nj}}{\left(\Delta f_{njm}\right)}^{2}-{\left(\bar{\Delta f_{nj}}\right)}^{2}\right)$$

(A.2) $$\Delta f_{njm}=f_{njm}-\sum_{r=1}^{R}s_{nr}e^{-\sum_{k=1}^{K}u_{k}(E_{r})\left(\sum_{t=1}^{T}x_{kt}p_{njtm}\right)}$$

(A.3) $$\bar{\Delta f_{nj}}=\frac{1}{M_{nj}}\sum_{m=1}^{M_{nj}}\left(\Delta f_{njm}\right)$$

where $\Delta f_{\mathrm{njm}}$, $M_{nj}$ and $f_{\mathrm{njm}}$ represent $\Delta f_{nm}$, $M_{n}$ and $f_{nm}$ of model (18) at the *j*-th angle of the *n*-th voltage, respectively. $p_{\mathrm{njtm}}$ represents the $p_{\mathrm{ntm}}$ of model (18) at the *j*-th angle of the *n*-th voltage.

The Lagrange form of model (23) is

(A.4) $$L(S_{n},E,X,A_{n},B,C,D)=G(S_{n},E,X)-tr(B^{T}E)-tr(C^{T}X)-\sum_{n=1}^{N}tr(A_{n}^{T}S_{n})$$

where $A_{n}=(a_{n1},a_{n2},\ldots ,a_{nR})$, $B={\left(b_{1},b_{2},\ldots ,b_{R}\right)}^{T}$ and $C={\left(c_{kt}\right)}_{KT}$. These are the slack variables and match $S_{n}$, **E** and *X*. *tr*(•) denotes the matrix trace. The KKT conditions are

(A.5) $$\frac{\partial L}{S_{n}}=0$$

(A.6) $$\frac{\partial L}{\partial E}=0$$

(A.7) $$\frac{\partial L}{\partial X}=0$$

(A.8) $$tr(A_{n}^{T}S_{n})=0$$

(A.9) $$tr(B^{T}E)=0$$

(A.10) $$tr(C^{T}X)=0$$

(A.11) $$A_{n}\geq 0,S_{n}\geq 0$$

(A.12) B≥0,E>0

(A.13) C≥0,X≥0

From formulas (A.9) and (A.12), we have

(A.14) B=0

The component form of (A.5) is

(A.15) $$\frac{\partial L}{S_{nr}}=\frac{\partial G}{\partial s_{nr}}-a_{nr}=0$$

Thus,

(A.16) $$s_{nr}\left(\frac{\partial G}{\partial s_{nr}}-a_{nr}\right)=s_{nr}\cdot 0=0$$

Substituting formula (A.8) into formula (A.16) yields

(A.17) $$s_{nr}\frac{\partial G}{\partial s_{nr}}=0$$

The detailed expression is

(A.18) $$s_{nr}\left(\sum_{j=1}^{J_{n}}\left(2\bar{\Delta f_{nj}}\left(\frac{1}{M_{nj}}\sum_{m=1}^{M_{nj}}\left(e^{-\sum_{k=1}^{K}u_{k}(E_{r})\left(\sum_{t=1}^{T}x_{kt}p_{njtm}\right)}\right)\right)\right)\right)-s_{nr}\left(\sum_{j=1}^{J_{n}}\left(2\frac{1}{M_{nj}}\sum_{m=1}^{M_{nj}}\Delta f_{njm}e^{-\sum_{k=1}^{K}u_{k}(E_{r})\left(\sum_{t=1}^{T}x_{kt}p_{njtm}\right)}\right)\right)=0$$

Substituting formulas (A.2) and (A.3) into formula (A.18) and combining like terms yields

(A.19) $$\begin{array}{c} s_{nr}\sum_{j=1}^{J_{n}}\sum_{m=1}^{M_{nj}}\left(\frac{1}{M_{nj}}\left(f_{njm}+\left(\frac{1}{M_{nj}}\sum_{m=1}^{M_{nj}}\left(\sum_{r=1}^{R}s_{nr}e^{-\sum_{k=1}^{K}u_{k}(E_{r})\left(\sum_{t=1}^{T}x_{kt}p_{njtm}\right)}\right)\right)\right)e^{-\sum_{k=1}^{K}u_{k}(E_{r})\left(\sum_{t=1}^{T}x_{kt}p_{njtm}\right)}\right)\\ =s_{nr}\sum_{j=1}^{J_{n}}\sum_{m=1}^{M_{nj}}\left(\frac{1}{M_{nj}}\left(\sum_{r=1}^{R}s_{nr}e^{-\sum_{k=1}^{K}u_{k}(E_{r})\left(\sum_{t=1}^{T}x_{kt}p_{njtm}\right)}+\left(\frac{1}{M_{nj}}\sum_{m=1}^{M_{nj}}\left(f_{njm}\right)\right)\right)e^{-\sum_{k=1}^{K}u_{k}(E_{r})\left(\sum_{t=1}^{T}x_{kt}p_{njtm}\right)}\right) \end{array}$$

Thus,

(A.20) $$s_{nr}=s_{nr}\frac{\sum_{j=1}^{J_{n}}\sum_{m=1}^{M_{nj}}\left(\frac{1}{M_{nj}}\left(\sum_{r=1}^{R}s_{nr}e^{-\sum_{k=1}^{K}u_{k}(E_{r})\left(\sum_{t=1}^{T}x_{kt}p_{njtm}\right)}+\left(\frac{1}{M_{nj}}\sum_{m=1}^{M_{nj}}\left(f_{njm}\right)\right)\right)e^{-\sum_{k=1}^{K}u_{k}(E_{r})\left(\sum_{t=1}^{T}x_{kt}p_{njtm}\right)}\right)}{\sum_{j=1}^{J_{n}}\sum_{m=1}^{M_{nj}}\left(\frac{1}{M_{nj}}\left(f_{njm}+\left(\frac{1}{M_{nj}}\sum_{m=1}^{M_{nj}}\left(\sum_{r=1}^{R}s_{nr}e^{-\sum_{k=1}^{K}u_{k}(E_{r})\left(\sum_{t=1}^{T}x_{kt}p_{njtm}\right)}\right)\right)\right)e^{-\sum_{k=1}^{K}u_{k}(E_{r})\left(\sum_{t=1}^{T}x_{kt}p_{njtm}\right)}\right)}$$

This is the iterative formula for $s_{nr}$. The matrix form is presented in formula (26). Similarly, the iterative formulas for $E_{r}$ and $x_{kt}$ are

(A.21) $$E_{r}=E_{r}\begin{array}{c} \frac{\sum_{k=1}^{K}\left(u_{k}^{\prime }(E_{r})\sum_{n=1}^{N}\sum_{j=1}^{J_{n}}\sum_{m=1}^{M_{nj}}s_{nr}\left(\frac{1}{M_{nj}}\left(\left(\sum_{r=1}^{R}s_{nr}e^{-\sum_{k=1}^{K}u_{k}(E_{r})\left(\sum_{t=1}^{T}x_{kt}p_{njtm}\right)}\right)+\left(\frac{1}{M_{nj}}\sum_{m=1}^{M_{nj}}\left(f_{njm}\right)\right)\right)\right)\left(\sum_{t=1}^{T}x_{kt}p_{jtm}\right)e^{-\sum_{k=1}^{K}u_{k}(E_{r})\left(\sum_{t=1}^{T}x_{kt}p_{njtm}\right)}\right)}{\sum_{k=1}^{K}\left(u_{k}^{\prime }(E_{r})\sum_{n=1}^{N}\sum_{j=1}^{J_{n}}\sum_{m=1}^{M_{nj}}s_{nr}\frac{1}{M_{nj}}\left(f_{njm}+\left(\frac{1}{M_{nj}}\sum_{m=1}^{M_{nj}}\left(\sum_{r=1}^{R}s_{nr}e^{-\sum_{k=1}^{K}u_{k}(E_{r})\left(\sum_{t=1}^{T}x_{kt}p_{njtm}\right)}\right)\right)\right)\left(\sum_{t=1}^{T}x_{kt}p_{jtm}\right)e^{-\sum_{k=1}^{K}u_{k}(E_{r})\left(\sum_{t=1}^{T}x_{kt}p_{njtm}\right)}\right)} \end{array}$$

(A.22) $$x_{kt}=x_{kt}\frac{\sum_{n=1}^{N}\sum_{j=1}^{J_{n}}\sum_{m=1}^{M_{nj}}\sum_{r=1}^{R}\frac{1}{M_{nj}}\left(\sum_{r=1}^{R}s_{nr}e^{-\sum_{k=1}^{K}u_{k}(E_{r})\left(\sum_{t=1}^{T}x_{kt}p_{njtm}\right)}+\left(\frac{1}{M_{nj}}\sum_{m=1}^{M_{nj}}\left(f_{njm}\right)\right)\right)s_{nr}u_{rk}p_{jtm}e^{-\sum_{k=1}^{K}u_{k}(E_{r})\left(\sum_{t=1}^{T}x_{kt}p_{njtm}\right)}}{\sum_{n=1}^{N}\sum_{j=1}^{J_{n}}\sum_{m=1}^{M_{nj}}\sum_{r=1}^{R}\frac{1}{M_{nj}}\left(f_{njm}+\left(\frac{1}{M_{nj}}\sum_{m=1}^{M_{nj}}\left(\sum_{r=1}^{R}s_{nr}e^{-\sum_{k=1}^{K}u_{k}(E_{r})\left(\sum_{t=1}^{T}x_{kt}p_{njtm}\right)}\right)\right)\right)s_{nr}u_{rk}p_{jtm}e^{-\sum_{k=1}^{K}u_{k}(E_{r})\left(\sum_{t=1}^{T}x_{kt}p_{njtm}\right)}}$$

Formulas (27) and (28) are the corresponding matrix forms of (A.21) and (A.22), respectively.
